# Supplementary material for: Machine learning enables improved runtime and precision for bio-loggers on seabirds
Source: Commun Biol. 2020 Oct 30;3:633. doi: 10.1038/s42003-020-01356-8 (PMC7603325; doi:10.1038/s42003-020-01356-8)
Supplement: Supplementary file 1 — Supplementary Information [file 42003_2020_1356_MOESM1_ESM.pdf]

Supplementary Information for:

Machine learning enables improved runtime and precision for bio-loggers on seabirds

Authors:

Joseph Korpela, Hirokazu Suzuki, Sakiko Matsumoto, Yuichi Mizutani, Masaki Samejima, Takuya Maekawa*, Junichi Nakai, Ken Yoda

***Correspondence:**

maekawa@ist.osaka-u.ac.jp

Biological background. In this paper, we have included two case studies for AIoA applications, in which we recorded the foraging and group behaviours of black-tailed gulls and streaked shearwaters, respectively.

Black-tailed gulls. Foraging is one of the most important behaviours exhibited by animals. Foraging behaviour has been intensively studied theoretically since the 1970s[1], but is still difficult to record in the field for various species (e.g., marine animals). Recently, animal-borne video cameras have become a promising candidate for recording the foraging behaviour of wild animals[2]. However, video loggers consume considerable battery power for recording videos, which limits their maximum recording time to several hours for birds with a body mass of 500 g[3]. While conventional methods such as continuous video recording might still capture occasional shots[4], such methods make inefficient use of their resources. In fact, previous studies have failed to film the foraging behaviour of black-tailed gulls at sea due to this limited battery life[5].

When foraging, animals perform a variety of movements. Given that accelerometers can be used to identify and categorize behaviour-specific movements of wild animals[6,7], e.g., food capture events, an accelerometer (low-cost sensor) can therefore be used to control a video camera (high-cost sensor), activating the camera when the target behaviour is detected. In this study, we recorded the foraging behaviour of omnivorous gulls, including previously unrecorded behaviour, using accelerometer-based AI to detect the gulls as they used specific foraging methods, such as surface-dipping and plunging.

Streaked shearwaters. Individual animals are expected to perform ARS in areas where they encounter more prey resources[8]. The patchily distributed prey may create a high degree of overlap in ARS zones among individuals, possibly causing group foraging[9,10], which may be an important strategy that provides benefits to the individuals[11]. However, this behaviour has been poorly documented among marine animals due to the limited battery life of video bio-loggers and the impracticality of attaching simpler GPS bio-loggers to all the individual animals that make up

the groups. By using GPS-based AI to capture videos of streaked shearwaters during ARS, we were able to test whether they forage in flocks during ARS, motivated by the flocks of hundreds to thousands of the species that are often observed at sea during their breeding season.

Training datasets. The data used to train the AIoA for the black-tailed gulls was collected in 2017 from five birds from the colony on Kabushima Island (40°32’19”N, 141°33’27”E; Aomori, Japan) using Axy-Trek bio-loggers (TechnoSmArt, Roma, Italy). These Axy-Trek bio-loggers were mounted on the animals’ backs when collecting data. The data was labelled to identify periods of possible foraging, flying, and stationary behaviour, with the goal of training a classifier that could differentiate between foraging and non-foraging behaviours based on acceleration data (see Fig. 3a for examples of acceleration data from foraging and non-foraging behaviours). Note that since no video was available when labelling the training data prior to deploying our bio-loggers, the ecologists were only able to label *possible* foraging events, identified based on dips in the acceleration data that correspond to short dives. However, each iteration of our experiments using AIoA should provide more ground truth information (i.e., video) to use when labelling, making more exact labelling possible in the future.

Along with the black-tailed gulls, we also evaluated our proposed method when used on streaked shearwaters from a colony on Awashima Island (38°27'57"N, 139°14'03"E; Niigata, Japan). The data used to train our models for the shearwaters came from data collected using Axy-Trek bio-loggers in 2016 from 31 birds at the Awashima colony. These Axy-Trek bio-loggers were mounted on the animals’ backs when collecting data. The shearwater data was labelled to identify periods of ARS, transit, and stationary behaviours, with the goal of training a classifier that could detect ARS behaviour based on GPS data (see *GPS features* for details). See Yoda et al. 2012[5] and Matsumoto et al. 2017[12] for general details about the field experiments.

a

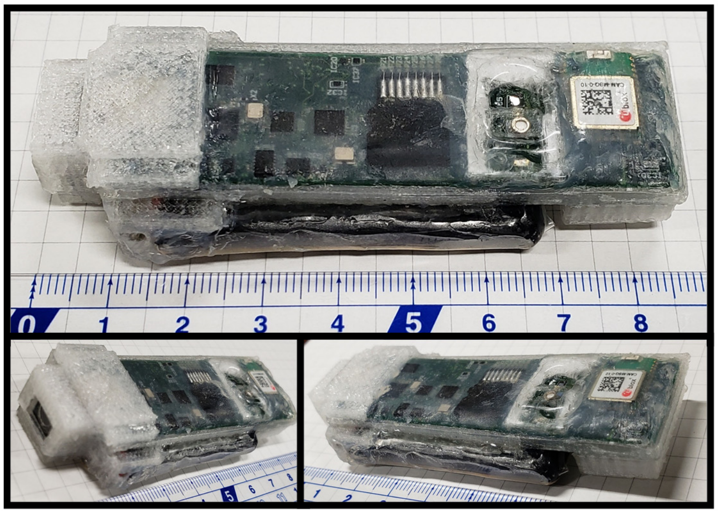

b

Sensor type	Model	Sampling rate
GPS	CAM-M8Q	1/60 Hz
Accelerometer	MPU-9250	25 Hz
Gyroscope	MPU-9250	25 Hz
Magnetometer	AK8963	1 Hz
Water pressure	MS5837-30BA	1 Hz
Barometric pressure	LPS22HB	1 Hz
Temperature	MS5837-30BA	1 Hz
Illumination	BH1721FVC	1 Hz

Supplementary Fig. 1 Video bio-logger hardware. a Images of one of the video bio-loggers used in this study. **b** A list of the low-cost sensors built into the bio-loggers used in this study.

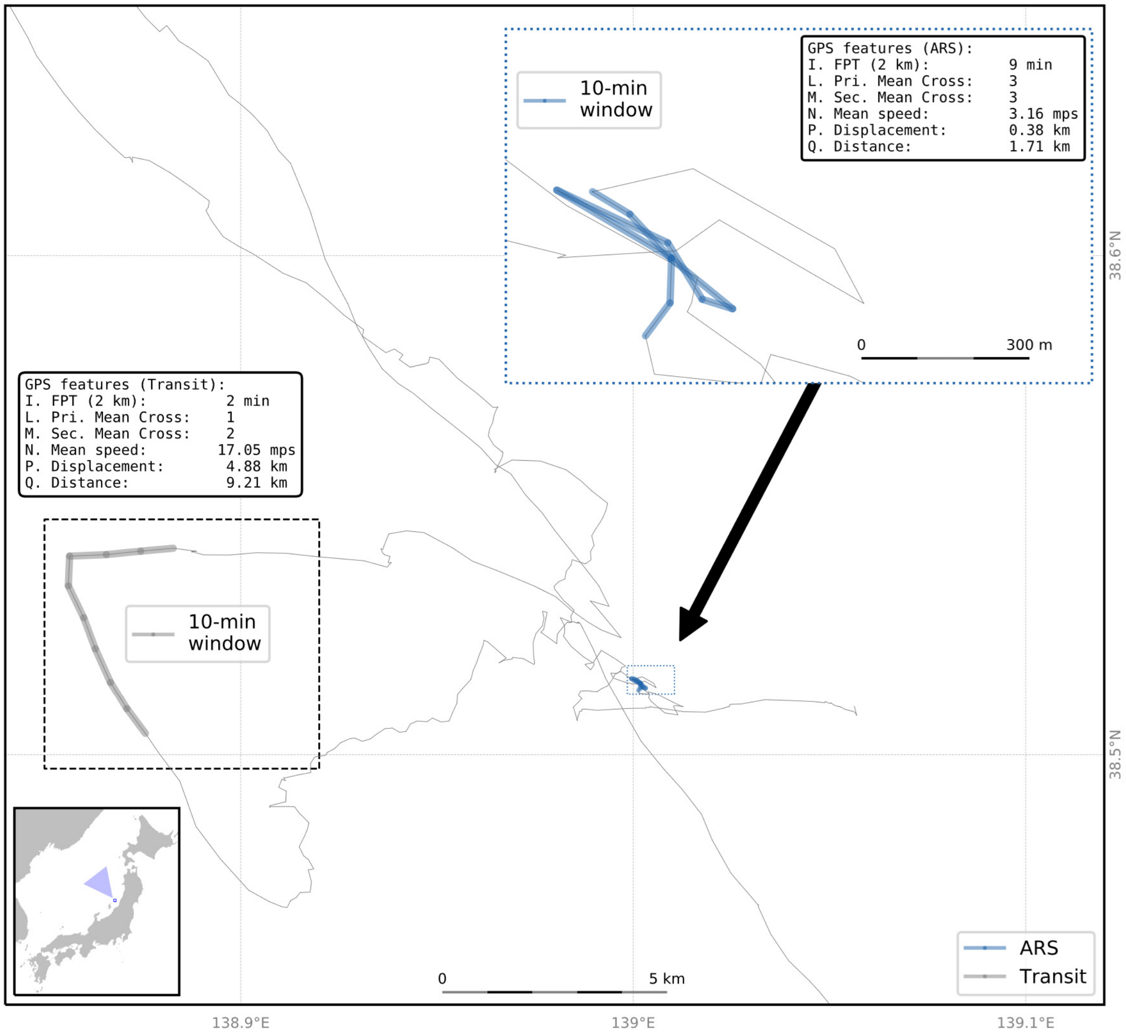

Supplementary Fig. 2 Examples of GPS-based features. This track shows GPS data collected from a streaked shearwater. Two sections of the track are highlighted, with the grey highlighted section capturing a 10-minute window of transit behaviour and the blue highlighted section capturing a 10-minute window of ARS behaviour. The two inset boxes list several GPS-based features extracted from each of these 10-minute windows, with the letters listed corresponding to the letters used in Supplementary Table 1.

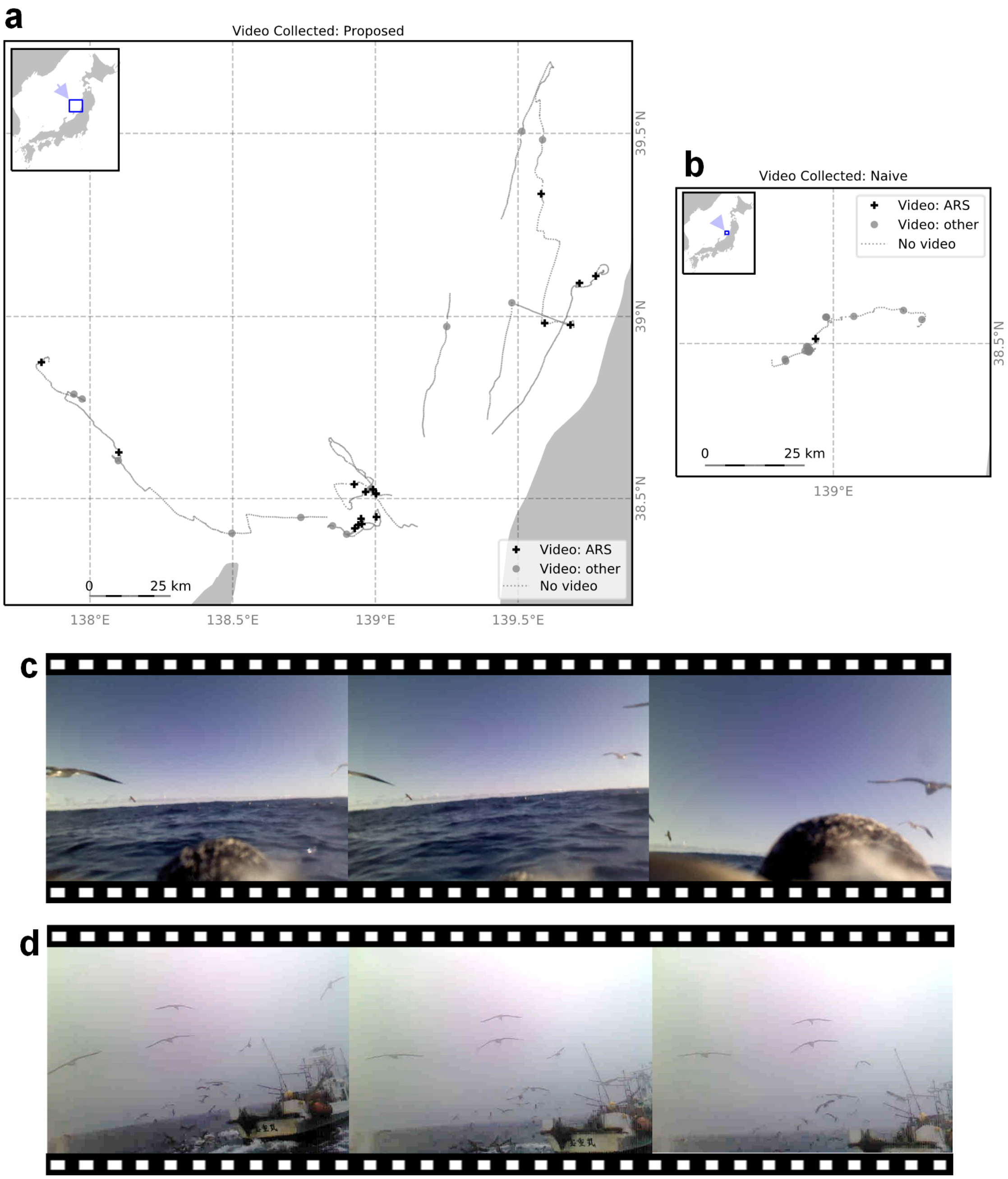

Supplementary Fig. 3 Results of AIoA-based video control for streaked shearwaters. a GPS tracks collected by bio-loggers using the proposed method, with the black markers representing videos of target (ARS) behaviour and the grey markers representing videos of non-target (transit and stationary) behaviour. **b** GPS tracks collected by bio-loggers using the naive method (periodic sampling), with the black markers representing videos of target behaviour and the grey markers representing videos of non-target behaviour. **c** Frames taken from video

captured using AIoA of a streaked shearwater performing ARS with other birds present in the images. **d** Frames taken from video captured using AIoA of a streaked shearwater performing ARS with a fishing boat and several other birds present in the images. Note: The brightness of the images in (**d**) has been increased by 40% from the original video frames.

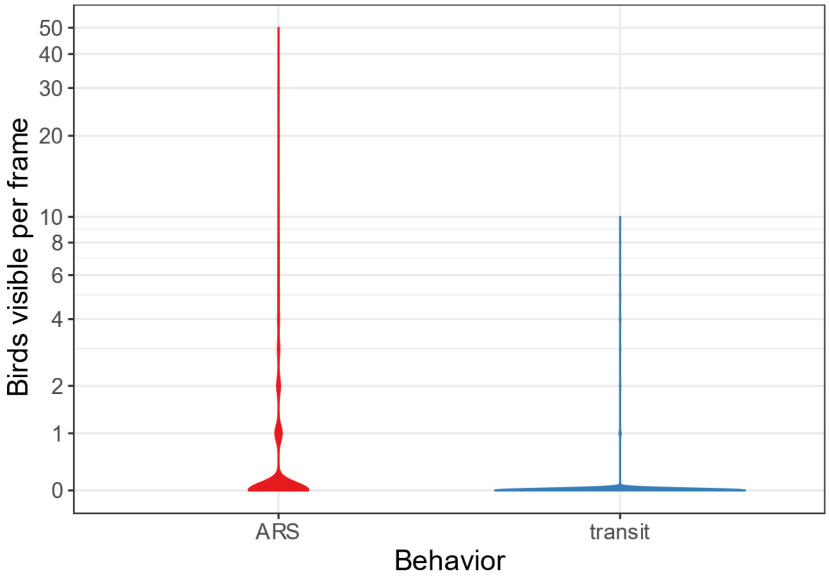

Supplementary Fig. 4 Distribution of the dataset used during analysis of group formation during ARS. This violin plot showing the distribution of the dataset used during GLMM analysis of the relationship between ARS behaviour and group formation for streaked shearwaters, with the y-axis shown in log(10) scale. The dataset is divided into two groups, with the 29,195 data points corresponding to ARS behaviour represented on the left and the 23,741 data points corresponding to transit behaviour represented on the right.

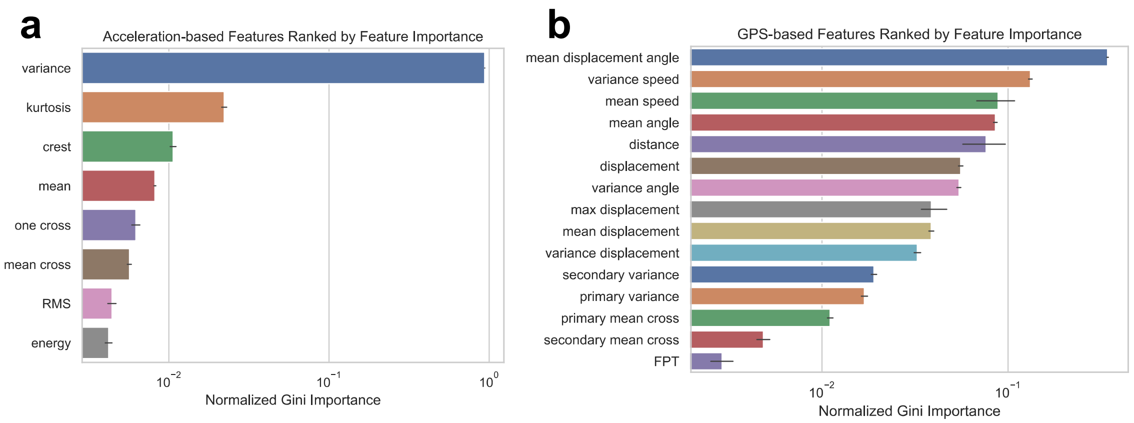

Supplementary Fig. 5 Features ranked by importance. a The acceleration-based features ranked by their Normalized Gini Importance (feature importance) with the error bars corresponding to the 95% confidence interval based on 10 iterations of decision tree generation. These values were computed using the tree module of Python's scikit-learn package (v.0.20.0) using the data described in *Training datasets*. **b** The GPS-based features ranked by their Normalized Gini Importance (feature importance) with the error bars corresponding to the 95% confidence interval based on 10 iterations of decision tree generation. These values were computed using the tree module of Python's scikit-learn package (v.0.20.0) using the data described in *Training Datasets*.

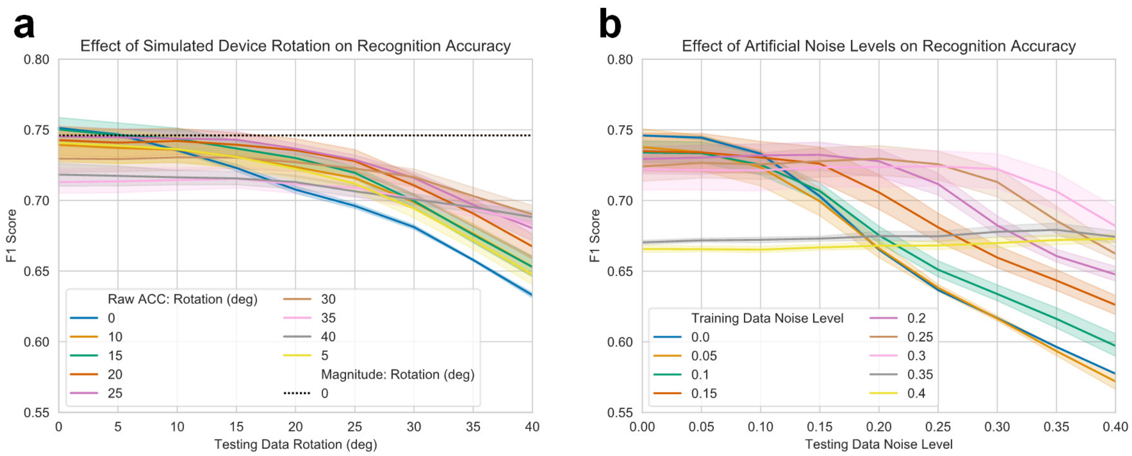

Supplementary Fig. 6 Improving the robustness of animal-borne AI. a Results from testing the effect of simulated device rotation on accuracy. Each solid line represents a training dataset in which the original dataset has been augmented with rotated data, while each testing dataset (x-axis) includes only rotated data. The values given for rotation indicate the maximum rotation applied to any axis, with each 1-second window rotated on each axis by a random amount up to that value. The shaded areas correspond to 95% confidence intervals based on running the test 10 times using 10 separately generated datasets. **b** Results from testing the effect of artificial noise on accuracy. Each line represents a training dataset in which the original dataset has been augmented with data that has artificial noise added, while each testing dataset (x-axis) includes only data that has noise added. The value (n) given for the artificial noise indicates the maximum amount of noise added to any 1-second window of data, with each window altered by multiplying all its original values by a random factor in the range [1-n, 1+n]. The shaded areas correspond to 95% confidence intervals based on running the test 10 times using 10 separately generated datasets.

Supplementary Table 1 Feature descriptions and estimated costs.

	Feature Name	Data Type	Estimated Size (Bytes)	Requires	Description
**A**	mean	ACC	40	*None*	Mean value.
**B**	mean cross	ACC	102	**A**	Count of how many times two adjacent values cross over the mean value. Gives an approximate measure of frequency.
**C**	one cross	ACC	94	*None*	Count of how many times two adjacent values cross over 1. Gives an approximate measure of frequency.
**D**	energy	ACC	60	*None*	Computed as the sum of squared discrete samples in the window divided by the number of samples in the window.
**E**	RMS	ACC	246	*None*	Root mean square.
**F**	kurtosis	ACC	680	*None*	The fourth standardized moment. Measures presence of extreme outlying values.
**G**	variance	ACC	210	*None*	Variance.
**H**	crest	ACC	240	**E**	Crest factor. Measures shape of wave peaks in the signal (sharp vs rounded).
**I**	FPT	GPS	144	**X**	First passage time. Time before displacement exceeds 2 km from first location in the window.
**J**	primary variance	GPS	0	**Y**	Variance in longitude values after running *rotation* (**Y**).
**K**	secondary variance	GPS	0	**Y**	Variance in latitude values after running *rotation* (**Y**).
**L**	primary mean cross	GPS	204	**Y**	Mean cross in longitude values after running *rotation* (**Y**).
**M**	secondary mean cross	GPS	204	**Y**	Mean cross in latitude values after running *rotation* (**Y**).
**N**	mean speed	GPS	108	**X**	Mean speed computed based on Manhattan distance.
**O**	variance speed	GPS	202	**X**	Variance in speed computed based on Manhattan distance.
**P**	displacement	GPS	6	**X**	Distance between the first and last locations in the window.
**Q**	distance	GPS	114	**X**	Sum of the distances between each location in the window.
**R**	max displacement	GPS	114	**X**	Maximum displacement between first location and any following location in the window.
**S**	mean displacement	GPS	338	**X**	Mean displacement from first location and all following locations in the window.
**T**	variance displacement	GPS	448	**X**	Variance in displacements between first location and all following locations in the window.
**U**	mean angle	GPS	112	**Z**	Mean of angles formed by all sets of three adjacent locations in the window.
**V**	variance angle	GPS	234	**Z**	Variance in angles formed by all sets of three adjacent locations in the window.
**W**	mean displacement angle	GPS	110	**Z**	Mean displacement angle for all locations in the window. Displacement angle is computed as the angle formed when using the first and last locations in the window as endpoints and any other location in the window as the midpoint.
**X**	distance	GPS	828	*None*	Helper function run to compute Manhattan distance between pairs of GPS coordinates.
**Y**	rotation	GPS	1,874	*None*	Helper function run to enable computation of other features. Rotates the coordinates window around their mean latitude and longitude values to maximize the variance in the longitudinal axis.
**Z**	angle	GPS	1,712	*None*	Helper function run to compute angles formed by sets of three GPS coordinates.

Each of the features extracted on board the bio-logger for use in AIoA. The data type *ACC* indicates features extracted from magnitude of acceleration values obtained from the acceleration sensor while *GPS* refers to features extracted from GPS data. The *Requires* column lists any features that must be extracted prior to extracting the feature listed in that row. For example,

feature **B** (*mean cross*) requires that you first compute feature **A** (*mean*). Features **X**, **Y**, and **Z** are helper functions that are only computed as a prerequisite for other GPS features, and are not directly used during classification.

Supplementary references

1. Charnov, E. L. Optimal foraging, the marginal value theorem. *Theor. Popul. Biol.* **9**, 129–136 (1976).
2. Rutz, C., Bluff, L. A., Weir, A. A. S. & Kacelnik, A. Video cameras on wild birds. *Science* **318**, 765 (2007).
3. Yoda, K. Advances in bio-logging techniques and their application to study navigation in wild seabirds. *Adv. Robot.* **33**, 108–117 (2019).
4. Handley, J. M. & Pistorius, P. Kleptoparasitism in foraging gentoo penguins Pygoscelis papua. *Polar Biol.* **39**, 391–395 (2016).
5. Yoda, K., Tomita, N., Mizutani, Y., Narita, A. & Niizuma, Y. Spatio-temporal responses of black-tailed gulls to natural and anthropogenic food resources. *Mar. Ecol. Prog. Ser.* **466**, 249–259 (2012).
6. Yoda, K. *et al.* A new technique for monitoring the behaviour of free-ranging Adelie penguins. *J. Exp. Biol.* **204**, 685–690 (2001).
7. Nathan, R. *et al.* Using tri-axial acceleration data to identify behavioral modes of free-ranging animals: general concepts and tools illustrated for griffon vultures. *J. Exp. Biol.* **215**, 986–996 (2012).
8. Kareiva, P. & Odell, G. Swarms of predators exhibit 'preytaxis' if individual predators use area-restricted search. *Am. Nat.* **130**, 233–270 (1987).
9. Weimerskirch, H., Bertrand, S., Silva, J., Marques, J. C. & Goya, E. Use of social information in seabirds: Compass rafts indicate the heading of food patches. *PLoS One* **5**, e9928 (2010).
10. Evans, J. C., Dall, S. R. X., Bolton, M., Owen, E. & Votier, S. C. Social foraging European shags: GPS tracking reveals birds from neighbouring colonies have shared foraging grounds. *J. Ornithol.* **157**, 23–32 (2016).
11. Giraldeau, L.-A. & Caraco, T. *Social Foraging Theory: Social Foraging Theory* (Princeton University Press, 2000). doi:10.2307/j.ctv36zrk6.5
12. Matsumoto, S., Yamamoto, T., Yamamoto, M., Zavalaga, C. B. & Yoda, K. Sex-related

differences in the foraging movement of streaked shearwaters Calonectris leucomelas breeding on Awashima Island in the Sea of Japan. *Ornithol. Sci.* **16**, 23–32 (2017).
