# Supplementary material for: Machine learning enables improved runtime and precision for bio-loggers on seabirds
Source: Commun Biol. 2020 Oct 30;3:633. doi: 10.1038/s42003-020-01356-8 (PMC7603325; doi:10.1038/s42003-020-01356-8)
Supplement: Supplementary file 3 — Description of Additional Supplementary Files [file 42003_2020_1356_MOESM3_ESM.pdf]

Description of Additional Supplementary Files

File Name: Supplementary Movie 1
Description: Intraspecific kleptoparasitism by a black-tailed gull. Video captured using AIoA of a black-tailed gull performing intraspecific kleptoparasitism while flying over the ocean near Aomori Prefecture, Japan.

File Name: Supplementary Movie 2
Description: Black-tailed gull foraging for fish. Video captured using AIoA of a black-tailed gull foraging for fish while flying over the ocean near Aomori Prefecture, Japan.

File Name: Supplementary Movie 3
Description: Black-tailed gull foraging for insects. Video captured using AIoA of a black-tailed gull foraging for insects while flying over the ocean near Aomori Prefecture, Japan.

File Name: Supplementary Movie 4
Description: Streaked shearwater group formation during area restricted search. Video captured using AIoA of a streaked shearwater participating in group formation while performing area restricted search over the ocean near Niigata Prefecture, Japan.

File Name: Supplementary Data 1
Description: Source data of Figs. 1, 2, and 3

File Name: Supplementary Software 1
Description: Code run on our bio-loggers, code to generate a low-cost decision tree, and hardware diagrams of the bio-loggers used in this study.
