# Supplementary material for: Machine learning enables improved runtime and precision for bio-loggers on seabirds
Source: Commun Biol. 2020 Oct 30;3:633. doi: 10.1038/s42003-020-01356-8 (PMC7603325; doi:10.1038/s42003-020-01356-8)
Supplement: Supplementary file 8 — Supplementary Software 1 [file 42003_2020_1356_MOESM8_ESM.zip › CodeAvailability/PinoutTranslated.pdf]

AVR Pin Name	Arduino Pin Name	Connection	Purpose	State		Recommended pinmode
				Default	When set	
PC6/RESET	RESET	Reset pin	Reset CPU (operated by external board/programmer)	High (pullup)	Low (connected to external GND)	N/A
PD0/RXD	0	External UART pin (TXD)	Serial communication w/ external hardware (e.g., PC)	Follow UART specifications		
PD1/TXD	1	External UART pin (RXD)				
PD2/INT0(RXD)	2	Camera UART (TXD)	Communication with camera (via SoftSerial)	Follow UART specifications		
PD3/INT1	3	RTC interrupt (INT)	Receives interrupt signals from the RTC	High	Low (interrupt)	Input (pullup)
PD4/PCINT20	4	SD card's CD	Controls SD card's CD	Follow SD library specifications		
PB6/XTAL1	None (XTAL connection)	No connection				
PB7/XTAL2	None (XTAL connection)	No connection				
PD5/PCINT21	5	LED	LED on/off	Low (Off)	High (On)	Output
PD6/PCINT22	6	GPS power/GPS I2C switch	Controls GPS power on/off and GPS I2C connect/disconnect	Low (Off/Disconnect)	High (On/Connect)	Output
PD7/PCINT23	7	Camera power (record)	Controls camera power on/off and record start/stop (recording control can also be done separately)	Low (Off/Stop recording)	High (On/Start recording)	Output
PB0/PCINT0(TXD)	8	GPS UART (RXD)	Communication with GPS (via SoftSerial)	Follow UART specifications		
PB1/PCINT1(RXD)	9	GPS UART (TXD)				
PB2/PCINT2	10	External TEST button	Controlled by TEST button on IF board	Low (No connection/not pressed)	High (TEST pressed)	Input (Hi-z: pulldown)
PB3/PCINT3/MOSI	11	SD/External SPI connection (MOSI)	Controls SD card's CMD, controls the programmer's SPI MOSI	Follow SD library specifications and ATMEL ICE/AVRDUDE programmer specifications		
PB4/PCINT4/MISO	12	SD/External SPI connection (MISO)	Controls SD card's DAT0, controls the programmer's SPI MISO			
PB5/PCINT5/SCK	13	SD/External SPI connection (SCK)	Controls SD card's CLK, controls the programmer's SPI CLK			
PC0/ADC0/PCINT8(TXD)	A0	Camera UART (RXD)	Communication with camera (via SoftSerial)	Follow UART specifications		
PC1/ADC1/PCINT9	A1	Battery	Measures battery's potential (uses AD converter)	Measure potential to estimate battery level		Use Arduino's analogRead()
PC2/ADC2/PCINT10	A2	SD card power	SD card power switch	Low (Off)	High (On)	Output
PC3/ADC3/PCINT11	A3	IF board high	Detects whether an external board is connected	Low (No board detected)	High (Board detected)	Input (Hi-z: pulldown)
PC4/ADC4/PCINT12/SDA	A4	I2C connection (SDA)	I2C communication with various sensors	Follow I2C specifications		
PC5/ADC5/PCINT13/SCL	A5	I2C connection (SCL)				
