# Supplementary material for: Machine learning enables improved runtime and precision for bio-loggers on seabirds
Source: Commun Biol. 2020 Oct 30;3:633. doi: 10.1038/s42003-020-01356-8 (PMC7603325; doi:10.1038/s42003-020-01356-8)
Supplement: Supplementary file 10 — Reporting Summary [file 42003_2020_1356_MOESM10_ESM.pdf]

nature research

Corresponding author(s): Takuya Maekawa

Last updated by author(s): Aug 31, 2020

Reporting Summary

Nature Research wishes to improve the reproducibility of the work that we publish. This form provides structure for consistency and transparency in reporting. For further information on Nature Research policies, see our Editorial Policies and the Editorial Policy Checklist.

Statistics

For all statistical analyses, confirm that the following items are present in the figure legend, table legend, main text, or Methods section.

n/a	Confirmed	
☐	☒	The exact sample size (n) for each experimental group/condition, given as a discrete number and unit of measurement
☒	☐	A statement on whether measurements were taken from distinct samples or whether the same sample was measured repeatedly
☐	☒	The statistical test(s) used AND whether they are one- or two-sided<br>*Only common tests should be described solely by name; describe more complex techniques in the Methods section.*
☐	☒	A description of all covariates tested
☐	☒	A description of any assumptions or corrections, such as tests of normality and adjustment for multiple comparisons
☐	☒	A full description of the statistical parameters including central tendency (e.g. means) or other basic estimates (e.g. regression coefficient) AND variation (e.g. standard deviation) or associated estimates of uncertainty (e.g. confidence intervals)
☐	☒	For null hypothesis testing, the test statistic (e.g. F, t, r) with confidence intervals, effect sizes, degrees of freedom and P value noted<br>*Give P values as exact values whenever suitable.*
☒	☐	For Bayesian analysis, information on the choice of priors and Markov chain Monte Carlo settings
☒	☐	For hierarchical and complex designs, identification of the appropriate level for tests and full reporting of outcomes
☐	☒	Estimates of effect sizes (e.g. Cohen's d, Pearson's r), indicating how they were calculated

Our web collection on statistics for biologists contains articles on many of the points above.

Software and code

Policy information about availability of computer code

Data collection	Data was collected using custom C++ code run on our sensor data loggers. Training data was labeled using a custom Window's application written in C#.
Data analysis	Python’s scikit-learn package (v.0.20.0) was used to generate standard decision trees, compute feature importances, and analyze classification accuracy. Custom Python code based on scikit-learn's (v.0.20.0) RandomForestClassifier was used to implement our algorithm. Fisher’s exact tests were done using the exact2x2 package (v. 1.6.3) of R (v. 3.4.3). The GLMM analysis was conducted using the lmerTest package (v. 2.0--36) of R (v. 3.4.3).

For manuscripts utilizing custom algorithms or software that are central to the research but not yet described in published literature, software must be made available to editors and reviewers. We strongly encourage code deposition in a community repository (e.g. GitHub). See the Nature Research guidelines for submitting code & software for further information.

Data

Policy information about availability of data

All manuscripts must include a data availability statement. This statement should provide the following information, where applicable:

- Accession codes, unique identifiers, or web links for publicly available datasets
- A list of figures that have associated raw data
- A description of any restrictions on data availability

The data from this study are available from the corresponding author upon reasonable request.

Field-specific reporting

Please select the one below that is the best fit for your research. If you are not sure, read the appropriate sections before making your selection.

- [ ] Life sciences
- [ ] Behavioural & social sciences
- [x] Ecological, evolutionary & environmental sciences

For a reference copy of the document with all sections, see nature.com/documents/nr-reporting-summary-flat.pdf

Ecological, evolutionary & environmental sciences study design

All studies must disclose on these points even when the disclosure is negative.

Study description	This study describes the use of artificial intelligence on sensor data loggers attached to wild seabirds.
Research sample	Movements of individual Calonectris leucomelas (n = 8) were tracked from a colony located on Awashima Island in Niigata Prefecture, Japan. Movements of individual Larus crassirostris (n = 13) were tracked from a colony located on Kabushima Island near Hachinohe City, Japan.
Sampling strategy	Sample size was determined by the time available for deployment and the availability of sensor data loggers. The birds were captured alive at their nests by hand prior to logger deployment and subsequent release. Loggers were fitted externally within 10 minutes. Logger deployment was undertaken by the ecologists participating in this study with logger deployment procedures approved by institutional ethical boards.
Data collection	Data was collected using custom C++ code run on our custom sensor data loggers.
Timing and spatial scale	Data was collected between 2018 and 2019.
Data exclusions	Data from loggers that suffered hardware failures (e.g., due to the failure of the waterproofing material used on some loggers) were excluded. We have not yet analyzed the data collected by the proposed method in 2019.
Reproducibility	No experiments as such were conducted, rather our data are based on tracked movements of individual birds.
Randomization	The ecologists selected individuals that met minimum weight requirements for logger attachment (for animal welfare) at random from the colonies.
Blinding	We did not consider blinding of the measurements.

Did the study involve field work? [x] Yes [ ] No

Field work, collection and transport

Field conditions	Sensor data loggers were deployed on Calonectris leucomelas from a colony located on Awashima Island in Niigata Precture, Japan, at night during fair weather. Sensor data loggers were deployed on Larus crassirostris from a colony located on Kabushima Island near Hachinohe City, Japan, in the morning during fair weather.
Location	Locations of sensor data deployment and subsequent tracks of the birds are detailed in the paper.
Access & import/export	Procedures used for habitat access were approved by institutional ethical review committees.
Disturbance	Disturbance to individual bird behavior was minimized through completion of logger deployment procedures within 10 minutes of capture and by selecting birds to meet minimum size requirement for logger attachment. All procedures were approved by institutional ethical review committees.

Reporting for specific materials, systems and methods

We require information from authors about some types of materials, experimental systems and methods used in many studies. Here, indicate whether each material, system or method listed is relevant to your study. If you are not sure if a list item applies to your research, read the appropriate section before selecting a response.

Materials & experimental systems

n/a	Involved in the study
☒	☐ Antibodies
☒	☐ Eukaryotic cell lines
☒	☐ Palaeontology and archaeology
☐	☒ Animals and other organisms
☒	☐ Human research participants
☒	☐ Clinical data
☒	☐ Dual use research of concern

Methods

n/a	Involved in the study
☒	☐ ChIP-seq
☒	☐ Flow cytometry
☒	☐ MRI-based neuroimaging

Animals and other organisms

Policy information about studies involving animals; ARRIVE guidelines recommended for reporting animal research

Laboratory animals	This study did not involve laboratory animals.
Wild animals	Details on how animals were captured and handled along with logger deployment techniques are provided in Yoda et al. 2012 and Matsumoto et al. 2017, which are cited in the Supplementary Information.
Field-collected samples	This study did not involve samples collected from the field.
Ethics oversight	All experimental procedures were approved by the Animal Experimental Committee of Nagoya University. Black-tailed gulls: the procedures were approved by the Agency for Cultural Affairs, Japan, and the Aomori Prefectural Government. Streaked shearwaters: the study was conducted with permits from the Ministry of the Environment.

Note that full information on the approval of the study protocol must also be provided in the manuscript.
